# The Binding Efficiency and Interaction of *Lactobacillus casei* Shirota Toward Aflatoxin B1

**DOI:** 10.3389/fmicb.2018.01503

**Published:** 2018-07-10

**Authors:** Winnie-Pui-Pui Liew, Zainuddin Nurul-Adilah, Leslie T. L. Than, Sabran Mohd-Redzwan

**Affiliations:** ^1^Department of Nutrition and Dietetics, Faculty of Medicine and Health Sciences, Universiti Putra Malaysia, Serdang, Malaysia; ^2^Department of Medical Microbiology and Parasitology, Faculty of Medicine and Health Sciences, Universiti Putra Malaysia, Serdang, Malaysia

**Keywords:** AFB1 binding efficiency, *Lactobacillus casei* Shirota, adsorption isotherm, scanning electron microscopy, morphology, serum AFB1

## Abstract

The use of probiotic as dietary approach to prevent exposure to food contaminant, aflatoxin B1 (AFB1) has greatly increased. Several studies found that AFB1 binding to the bacterial cell wall is strain-specific. Moreover, the interaction between AFB1 and bacterial cell wall is not well-understood, thus warrants further investigation. This research was conducted to assess the ability of *Lactobacillus casei* Shirota (Lcs) to bind AFB1 at different concentrations and to determine AFB1 binding efficiency of different Lcs cell components including live cell, heat-treated, and cell wall. In addition, the interaction between AFB1 and Lcs was also evaluated via scanning electron microscopy (SEM) and through an animal study. The binding of AFB1 by all Lcs cell components depends on the concentration of available AFB1. Among all Lcs cell components, the live Lcs cells exhibited the highest binding efficiency (98%) toward AFB1. Besides, the SEM micrographs showed that AFB1 induced structural changes on the bacterial cell surface and morphology including rough and irregular surface along with a curve rod-shaped. *In vivo* experiment revealed that Lcs is capable to neutralize the toxicity of AFB1 on body weight and intestine through the binding process. The animal’s growth was stunted due to AFB1 exposure, however, such effect was significantly (*p* < 0.05) alleviated by Lcs. This phenomenon can be explained by a significant (*p* < 0.05) decreased level of blood serum AFB1 by Lcs (49.6 ± 8.05 ng/mL) compared to AFB1-exposed rats without treatment (88.12 ± 10.65 ng/mL). Taken together, this study highlights the potential use of Lcs as a preventive agent against aflatoxicosis via its strong binding capability.

## Introduction

Mycotoxin, a low-molecular-weight secondary metabolite produced by certain fungi is a highly toxic substance (Flores-Flores et al., 2015). The production of mycotoxin due to fungal infection in foods occurs mainly in tropical regions, with conditions such as high temperatures and moisture, unseasonal rains during harvest, and flash floods. Besides, other factors such as poor harvesting practices, improper storage, and inadequate optimal conditions during transportation, marketing and processing can also contribute to the growth of fungi in the food commodities and subsequently the production of mycotoxin (Umereweneza et al., 2018). Due to the incessant and ubiquitous exposure of mycotoxins, the contamination of food and agricultural commodities is a public concern globally, where their occurrence in the food chain cannot be disregarded. In fact, the dosage, duration of exposure, type of mycotoxins, and physiological, genetic and nutritional status (Antonissen et al., 2014) can influence the adverse effects of mycotoxins on human and animals health.

The Joint FAO/WHO Expert Committee on Food Additives (JECFA) described that several species of *Aspergillus* are the producer of aflatoxin B1 (AFB1), the predominant and most dangerous mycotoxin. In addition, a study conducted by the World Health Organisation (WHO) Foodborne Disease Burden Epidemiology Reference Group from 2007 to 2015 revealed that aflatoxin exposure was extremely high in the Western Pacific regions, which resulted in a median death rate of 1 per 200,000 inhabitants (WHO, 2015). Of many aflatoxin metabolites, the International Agency for Research on Cancer (IARC Working Group on the Evaluation of Carcinogenic Risks to Humans, 2002) has classified AFB1 as a Group 1 carcinogen (carcinogenic to humans and animals). It is evident that the carcinogenicity of aflatoxins is linked to the genotoxic mechanism of action that involves; (1) the metabolic activation of cytochrome P-450 (CYP) enzyme systems on a genotoxic epoxide (AFB1-8,9-epoxide) metabolite, (2) the formation of DNA adducts, and (3) the modification of the TP53 gene. Besides the main mutation of G:C→T:A at TP53 gene, Chawanthayatham et al. (2017) found a more heterogeneous set of mutations emerged during a tumor outgrowth induced by AFB1. AFB1 also causes several health problems including gastrointestinal pain, diarrhea, stunted growth, immunosuppressive, and neurotoxic effects in livestock and humans being, as evidenced by several studies (Bahey et al., 2015).

Several methods have been developed to reduce aflatoxin contamination, which includes enterosorption and chemoprotection methods. Enterosorption uses clay to adsorb tightly and selectively bind aflatoxin in the gastrointestinal tract, and subsequently reducing its bioavailability and associated toxicity effects. Nevertheless, clay comprised of chemicals which may pose risks to consumers (Yazdanpanah and Eslamizad, 2015). In contrast, chemoprotection method uses chemical agents (e.g., phenolic antioxidants) or dietary components (e.g., green tea polyphenols) in order to protect against the initiation or progression of aflatoxin-induced carcinogenesis. This strategy, however, is expensive thus impractical for the poor communities (Gorran et al., 2013). Recently, there has been increasing focus on alternative measures and affordable biological methods such as probiotic as one the dietary approaches to prevent aflatoxin exposure. Probiotics are defined as “live microorganisms that, when administered in adequate amounts, confer a health benefit on the host” (Hill et al., 2014). Probiotic can remove AFB1 via adsorption (Luo et al., 2018) or degradation (Adebo et al., 2017) mechanisms. Biodegradation of AFB1 is permanent yet requires longer duration compared to bioadsorption (Adebo et al., 2017). Besides, biodegradation modified structure of AFB1 and resulting in undesirable metabolites (example: aflatoxicol) which may be harmful to the host (Verheecke et al., 2016). Bioadsorption, on the other hand, involves a direct binding of AFB1 in a short period of time. Biological binding seems promising but AFB1 may be easily released and depends on the affinity of probiotic toward AFB1 (Luo et al., 2018). Lactobacilli have become the focus of intensive international research for their health-promotion effects in lactose intolerance, allergies, diarrhea, central nervous system function, and disorders, cholesterol reduction, eczema, immune function, and infections. Interestingly, this genus of bacteria is also found as an effective aflatoxin-reducing microorganism. It has been suggested that AFB1 is able to bind to the surface components of the cell wall of probiotic bacteria (Ahlberg et al., 2015). However, the binding mechanisms are not well studied and required further investigation.

In this study, probiotic *Lactobacillus casei* Shirota (Lcs) is used for the AFB1 detoxification purpose. In 1935, Lcs was found by Minoru Shirota and started selling as a probiotic dairy product, Yakult^TM^. Lcs has self-affirmed generally recognized as safe status and well-known for its health benefits in gastrointestinal problems as supported by extensive scientific research and findings (Kwan et al., 2017). Most of the research carried out using Lcs focus on its alleviation of gut diseases via immunomodulation, lactic acid production, and competitive inhibition toward pathogens. The first study on the AFB1 binding ability by Lcs was conducted by El-Nezami et al. (1998). However, the mechanism underlying the AFB1 binding by probiotics is not well understood. Therefore, in this study, *in vitro* experiments were conducted to determine the ability of Lcs cell components (live cell, heat treated cell, and cell wall fractions) to bind AFB1 via adsorption isotherms and to observe the binding of AFB1 by Lcs via scanning electron microscopy (SEM). For the *in vivo* experiment, a rat model was used to investigate the binding ability of Lcs as well as the effects of Lcs on body weight (b.w.) and histopathology of intestines.

## Materials and Methods

### Reagents and Apparatus

The media used for bacterial culture was Man deRogosa (MRS) broth (Himedia, India). Chemicals and reagents intended for AFB1 removal assay were pure AFB1 (Trilogy Analytical Laboratory Inc., United States) and activated carbon (Ultracarbon, Merck, Germany). For SEM analysis, materials required were glutaraldehyde (Sigma-Aldrich Company, United States), sodium cacodylate (Fisher Scientific, United States), osmium tetroxide (Fisher Scientific, United States), and acetone (Sigma-Aldrich Company, United States). For histology, materials such as paraformaldehyde, ethanol, haematoxylin, and eosin were purchased from Sigma-Aldrich Company, United States. A pure culture of bacteria Lcs was isolated from Yakult^®^ cultured drink, and the identity was confirmed as *L. casei* using 16s RNA sequencing service (First BASE Laboratories Sdn. Bhd, Malaysia).

### Preparation of Lcs Cell Components

*Lactobacillus casei* Shirota (Lcs) was cultured in MRS broth at 37°C for 18 h. For subsequent experiments, Lcs was used after three successive cultures. The concentration of bacteria was standardized at O.D. 1.0 (600 nm) using UV–VIS spectrophotometer (UV-1800, Shimadzu, Japan) and measured at 10^9^ cells on the MRS agar plate. After centrifugation (5417, Eppendorf, Germany) at 3500 rpm, 15 min, the pellet containing live cells was re-suspended in the same volume of PBS 1 × buffer (pH 7.4). All centrifugation was done at 3,500 rpm, 15 min at r.t. (25°C) unless mentioned otherwise. Lcs culture was separated into three different cell components such as bacterial live cells, heat-treated bacterial cell, and cell wall extracts.

Heat-treated bacterial cells were prepared by boiling the pellet containing live cells at 100°C, 1 h (Haskard et al., 2001). On the other hand, the cell wall extracts were prepared as described by Pérez-Martín et al. (2012), by sonicating the cells for 20 min, with the pulse control mode set on 0.5 and the amplitude of 70%, using a sonicator probe UP50H (Hielscher GMBH, Germany). After centrifugation (5,000 rpm, 15 min) the cell wall fractions were obtained. The bacterial pellet was then suspended in the same volume of PBS 1 × buffer.

#### *In Vitro* AFB1 Binding Assay

The evaluations of AF binding were performed according to Pizzolitto et al. (2011) with some modifications. The cell extracts and activated carbon (2 μg; positive control) were incubated with different concentrations of AFB1 (2, 4, 6, 8, and 10 μg/mL). The mixture was kept in an incubator for 1 h at 37°C and then centrifuged at 3,500 × *g* for 15 min. The supernatant was collected and used for AFB1 analysis. The concentration of AFB1 was assessed using enzyme-linked immunosorbent assay (ELISA) kits (Cusabio Biotech, China) based on the manufacturer’s instructions. The percentage of AFB1 binding in all assays was calculated by using the following equation:

$$\%\text{ of AFB1 binding=}\frac{\text{AFB1}(initial)-AFB1\left(\text{final}\right)}{\text{AFB1}\left(\text{final}\right)}\times 100\%,$$

The AFB1 concentration up to 10 μg/mL is relevant to human exposure, especially for populations with high prevalence of aflatoxin exposure in foods. The limits set by most countries for AFB1 and total aflatoxins are 5 and 20 mg/kg, respectively (Shim et al., 2014). The incubation period of 1 h was applied in this study to reflect the transit time in the human small intestine (McClements and Li, 2010) as the small intestine is the main site of aflatoxin absorption (Dogi et al., 2017).

##### Efficiency parameters

The interaction between Lcs and AFB1 was further studied by using a model developed by Bueno et al. (2007). The model explains the adsorption phenomenon to the microorganism surface. The relationship between the amounts of AFB1 adsorbed at the microorganism surface as a function of its solution concentration is described by an adsorption isotherm. The graph shows linearity at low values of AFB1 and then transitioning to a plateau. This type of isotherm can be described by the following equation:

Qe=M[KeqCe/(1+KeqCe)],

Where Qe (mg/g) is the amount of toxin per unit weight of adsorbent in adsorbing equilibrium; Ce (mg/L) is the equilibrium concentration of toxin; *M* is the maximum number of adsorption sites per microorganism; and Keq (expressed in liters per mole) is equivalent to the affinity of aflatoxin molecules for the adsorption sites. Moreover, extrapolation of these values was used to construct a graph of Ce/Qe versus Ce, where the efficiency of microorganism as AFB1 adsorbent can be determined through the slope 1/M and intercept of 1/KeqM (Sangsila et al., 2016). It is postulated that different strain of probiotic bacteria might have a different number of binding sites of AFB1, affinity, and efficiency.

##### Scanning electron microscopy (SEM)

Surface characterization of both control Lcs cells and AFB1 adsorbed cells was carried out using variable pressure SEM (VPSEM, LEO 1455, Leo Electron Microscopy Ltd, Germany). A total of 10^9^ cells were mixed in 1 mL of PBS (pH 7.2) that contained 2 μg/mL of AFB1 and incubated for 1 h at 37°C. The electron microscopic study was done using a method as described by Oslan et al. (2017). In brief, 2.5% (v/v) glutaraldehyde buffer was used to fix the samples for 4 h at 4°C. Then, the fixed cells were washed thrice for 10 min using 0.1 M sodium cacodylate buffer. After post-fixation in 1% (w/v) osmium tetroxide for 2 h at 4°C, the cells were subjected to washing before dehydration with a series of increasing concentrations of acetone (35–100%). The cell suspensions were incubated for 10 min in each acetone solution except for the 100% acetone, which was 15 min with three changes of acetone. Later, the samples were processed under a Critical Point Drying (CPD) method using Leica EM CPD 030 (Leica Microsystem, Germany). The samples were sputtered by a thin layer of gold using a sputter coater (SC 1620, Quarum, United Kingdom) then examined using VPSEM attached with energy dispersive X-ray analysis (EDX).

#### *In Vivo* AFB1 Binding Assay

##### Animals

Male Sprague Dawley (SD) rats (7–8 weeks old, 250–300g, *n* = 24) were obtained from Animal Resource Unit, Department of Veterinary Pathology and Microbiology, Faculty of Veterinary Medicine, University Putra Malaysia (UPM). This study was performed at animal research house of Comparative Medicine and Technology Unit (COMeT), Institute of Bioscience UPM. Two/three rats were housed in a cage with saw dust bedding. The rats were acclimatized under standard laboratory conditions [12 h light/night cycles (light: 0700–1900 h), 20–22°C, 1-week] prior to the AFB1 exposure and treatments. All rats were given *ad libitum* access to food and water during acclimatization period. Weight and feed intake were measured and monitored on weekly basis. The animal study was approved by the Institutional Animal Care and Use Committee UPM (UPM/IACUC/AUP-R098/2016).

##### Experimental study

Rats were randomly divided into three groups (*n* = 8) and subjected to AFB1 exposure except the control, group A: animals were oral gavaged with PBS 1 × buffer (pH 7.4) only; Treatment group B: the rat was supplemented with Lcs (10^9^ CFU) daily by oral gavage daily. Upon 5 days of probiotic supplementation, a complete dosage of 25 μg AFB1/kg b.w. was given to the rats of group B and group C via oral gavage. Group C was treated with AFB1 only. The treatments were carried out for 5 days per week (Qian et al., 2014).

During the treatment, Lcs was oral gavaged 4 h before the rats were gavaged with AFB1. The interval time of 4 h was applied in this study to reflect the time needed for the probiotic to reach the small intestine (Hardy, 1989) and to prevent the binding of AFB1 and Lcs occurs before entering the body of rats. The dosage of AFB1 in this study was selected based on the AFB1 level (30–450 ng/mL) present in food of developing countries (Daniel et al., 2011). Throughout the experiment, food and water were supplied *ad libitum* for the rats. B.w. of rats from all groups were recorded on weekly basis using electronic balance (A&D Co., Ltd., Tokyo, Japan). At the end of 4 weeks treatment, rats were anesthetized using ketamine and xylazine. Blood withdrawal was performed by cardiac puncture.

##### Collection of urine, blood, and organs

AFM1, a common AFB1 derivative found in the urine was measured. For AFM1 quantification assay, urine was collected using metabolic cage, a day before the rats were sacrificed. Upon scarification, about 5 ml blood was drawn from the artery vessel and transferred into EDTA vacutainers. Blood serum were separated using Kubota 2810 centrifuge (Japan) at 4°C for 13 min at 2000 × *g*. The serum was collected and stored at -80°C until further analysis for level of AFB1. For histological analysis purpose, small intestine and colon were collected and subjected to washing in PBS 1x (pH 7.4) before formaldehyde fixation.

##### Determination of urinary AFM1 and serum AFB1

Using ELISA kit, the concentration of AFM1 was determined (Helica Biosystems, Inc., United States) according to Mohd Redzwan et al. (2012). AFB1 in blood serum was measured using ELISA kit (RIDASCREEN, R-Biopharm, Germany) according to the manufacturer’s instructions.

##### Histopathological examination

The whole small intestine and colon was obtained from each rat and fixed in formalin solution 10%, Neutral Buffered (R&M Chemicals, United Kingdom) up to 3 days at room temperature (25°C). The fixed tissue samples were subjected to washing for several times using 80–95% ethanol. The tissue was then dehydrated in absolute ethanol before immersed in xylene for clearing and embedded in paraffin. Using microtome, the paraffin-embedded tissues were sectioned serially at 4 μm thickness. For qualitative histological analysis, the sections were routinely stained with hematoxylin and eosin (H&E) using automated slides stainer (Prisma-E2S, Sakura Seiki Co., Ltd., Japan). The microscopic viewing of stained tissue sections was blinded endpoint analysis.

### Statistical Analysis

All samples were analyzed with three replicates. For *in vitro* binding and efficiency parameters, the statistical differences between control and treatment groups (live cell, heat treated cell, and cell wall fractions) were analyzed by one-way analysis of variance and Tukey’s *post hoc* test using Minitab 18 for Windows. The serum AFB1 and urinary AFM1 levels in rats treated with AFB1 (group B and C) were analyzed by Independent *T*-Test using Minitab 18 for Windows. Significance difference was set at *p*-value < 0.05.

## Results and Discussion

### *In Vitro* AFB1 Binding Assay

**FIGURE 1:**
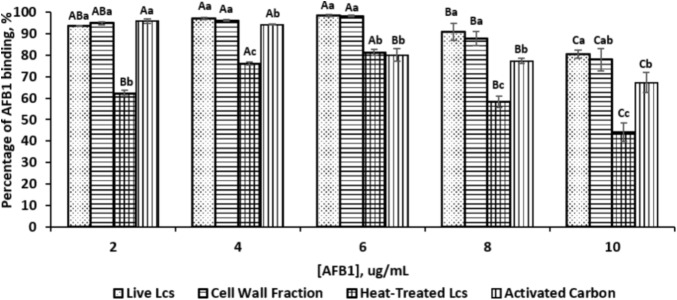
Percentage of AFB1 binding by different Lcs cell components and activated carbon at different concentration of AFB1. Each column represents different samples: dotted, live Lcs; horizontal lines, cell wall fraction; grid, heat-treated Lcs; vertical lines, activated carbon. Aliquots of 1 ml of 10^9^ cells (Lcs live cells; heat-treated cell; cell wall component) and activated carbon were suspended in PBS in the presence of AFB1 at the following concentrations: 2, 4, 6, 8, and 10 μg/mL. Upon incubated for 1 h at 37°C, samples were subjected to centrifugation. The supernatant was collected for unbound AFB1 analysis by ELISA. Data are means from triplicate experiments (error bars indicate mean ± SD). Means between different Lcs cell components and activated carbon within the same concentration with different lowercase letters (a, b, c) are significantly different (*p* < 0.05); Means between different concentration of the same Lcs cell component as well as activated carbon with different uppercase letters (A, B, C) are significantly different (*p* < 0.05).

The AFB1 binding properties of the three different Lcs cell components (live cell, heat treated cell, and cell wall fractions) are depicted in **Figure 1**. All of the Lcs cell components were capable to remove AFB1 in PBS, with varying removal abilities across different cell components as well as different AFB1 concentrations. In this study, an initial AFB1 concentration range from 2 to 10 μg/mL was tested. Both Lcs cell wall fractions and live cells showed the highest binding capacity at the AFB1 concentration of 6 μg/mL, with AFB1 binding capacity of 97 and 98% respectively. For the positive control, the highest binding capacity of 96% was reached at AFB1 concentration of 2 μg/mL, whereas, the maximum binding capacity for heat-treated Lcs cells was 81% at AFB1 concentration of 6 μg/mL. Similarly, Apás et al. (2014) found approximately a concentration of 2 × 10^10^ CFU/mL of *Bifidobacterium longum* and *Lactobacillus acidophilus* were capable of reducing AFB1 level to <0.1% and 13%. Another study using Lcs found a spontaneous reduction of 67% of AFB1 level when incubating 5 μg/mL AFB1 with approximately 1 × 10^10^ CFU/ml of Lcs live cells (El-Nezami et al., 1998). Nevertheless, Hernandez-Mendoza et al. (2009a) showed that the percentage of AFB1 bound by Lcs was approximately 30% at AFB1 concentration of 4.6 μg/mL after 4 h of incubation at 37°C. The difference in incubation period could be the factor which affect the binding capacity of Lcs obtained from these studies (El-Nezami et al., 1998; Hernandez-Mendoza et al., 2009a; Apás et al., 2014). The incubation period of 1 h was applied in the present study to reflect the transit time in human small intestine (McClements and Li, 2010). Besides, the absorption of aflatoxin mostly occurs in the small intestine (Dogi et al., 2017). In fact, incubation time plays a role in the binding efficiency as revealed by Zhao et al. (2015) on another mycotoxin, zearalenone. A longer incubation time affects the binding efficiency of *Lactobacillus plantarum* strains toward mycotoxin by decreasing the binding capacity (Zhao et al., 2015).

An increasing trend of binding capacity was observed from the AFB1 concentration of 2–6 μg/mL for all Lcs cell components. However, the binding capacity started to decrease at AFB1 concentration of 8 μg/mL. At the initial AFB1 concentration of 4 and 6 μg/mL, the binding capacity of both Lcs live cells and cell wall fractions was significantly (*p* < 0.05) higher compared to positive control and heat-treated cells. Meanwhile, the heat-treated Lcs cells demonstrated a significantly (*p* < 0.05) lower binding capacity at all concentrations compared to Lcs live cells, cell wall fractions, and positive control except at the AFB1 concentration of 6 μg/mL. The observation in this study was in line with the findings shown by Rahnama Vosough et al. (2014), in which the binding capacity of *Lactobacillus rhamnosus* GG was affected by the concentration of AFB1. The range of AFB1 concentration up to 10 μg/mL tested in this study is to reflect AFB1 exposure in the foods. Perhaps, different AFB1 concentration may have different outcomes and warrant further investigation.

#### Efficiency Parameters

**FIGURE 2:**
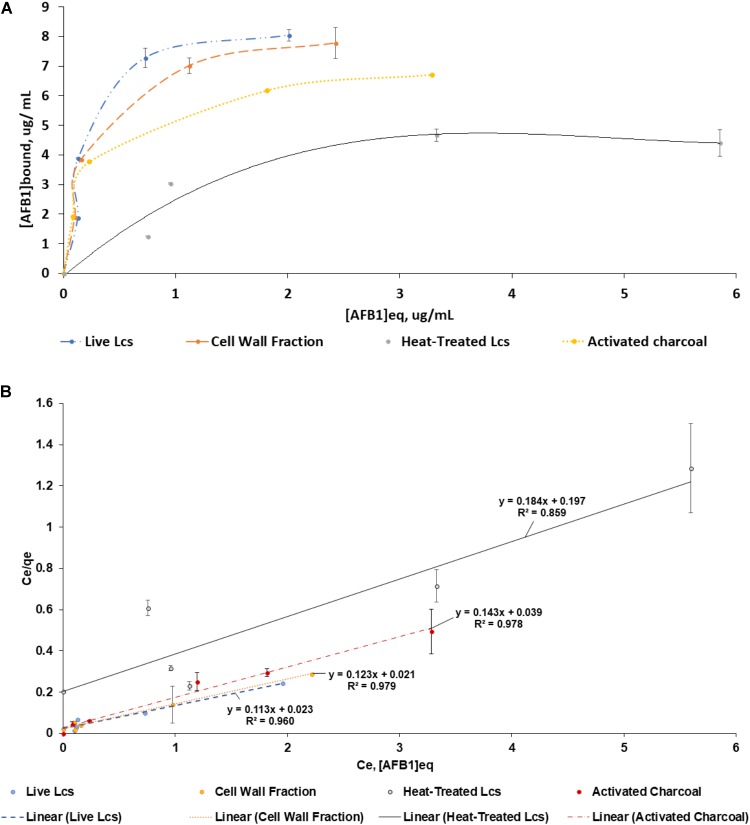
Adsorption isotherms of AFB1 by Lcs cell components. Aliquots of 1 ml of 10^9^ cells (Lcs live cells; heat-treated cell; cell wall component) and 2 μg of activated charcoal were suspended in PBS in the presence of AFB1 at the following concentrations: 2, 4, 6, 8, and 10 μg/mL. Then, the bacteria were incubated for 1 h at 37°C and pelleted by centrifugation. The supernatant was collected for free AFB1 analysis by ELISA. AFB1 bound to cells was calculated as the difference between the total AFB1 and the amount of free AFB1. **(A)** Saturation curve. **(B)** Ce vs. Ce/Qe of the same data as **(A)**. Data are means from triplicate experiments (error bars indicate mean ± SD).

A best-fit line was used to construct saturation curve of AFB1 by Lcs cell components (**Figure 2A**). In general, the curve increased steadily at the beginning and shifted to a plateau at an increasing AFB1 concentration. This type of isotherm can be described by the Langmuir isotherm, which is one of the most commonly used equations. The Langmuir equation is valid for a monolayer adsorption onto a surface with a fixed number of identical sites (Langmuir, 1918). The linearized form of the isotherm is obtained by plotting a graph of Ce vs. Ce/Qe, as shown in **Figure 2B**, where the Keq and *M* value of each Lcs cell components can be determined. Although the capability of Lcs to bind AFB1 in *in vitro* (Hernandez-Mendoza et al., 2009a) and *in vivo* (Mohd Redzwan et al., 2016) have been vastly reported, there is no data published on the AFB1 binding efficiency parameters of probiotic Lcs. In the present study, the live Lcs cells and cell wall components had the highest binding efficiency toward AFB1 (**Table 1**). Moreover, the Lcs live cells and cell wall fractions also showed significantly (*p* < 0.05) higher Keq value compared to the heat-treated cell. The Keq value describes the interacting force between the mycotoxin and microorganism cell wall. The high value of Keq of both Lcs live cells and cell wall fractions indicated a strong AFB1 binding process. Such phenomenon is favorable, as less AFB1 is released during the passage through the intestinal tract after bound to Lcs. Further analysis was conducted to determine efficiency of activated charcoal used in this experiment. For activated charcoal, the value obtained for total binding site, Keq, and efficiency were 2.24 ± 0.10 × 10^7^ site/g, 3.82 ± 0.94, and 8.62 ± 2.48 × 10^7^ respectively.

**Table 1:** Total binding sites per microorganism (M), equilibrium constant (Keq) and efficiency (M × Keq) for different Lcs cell components. M, Keq and M × Keq for different Lcs cell components were calculated by linear regression as described in **Figure 2B**.

Groups	M (1 x 10^10^ site/ cell)	Keq (1 x M^-1^)	Efficiency (1 x 10^10^)
Cell Wall	2.61 ± 0.11^a^	6.23 ± 1.49^a^	16.34 ± 4.58^a^
Live Cell	2.84 ± 0.16^a^	5.01 ± 1.06^a^	14.32 ± 3.85^a^
Heat-Treated	1.76 ± 0.23^b^	1.00 ± 0.26^b^	1.79 ± 0.70^b^


*^*1*^Results are expressed as mean ± standard deviation of mean (*n* = 3). Means between different Lcs cell components within the same efficiency parameter with different superscript letters (a and b) are significantly different (*p* < 0.05).*

Similar to other mycotoxins, AFB1 was found binding at the surface of Lactobacillus strains (*L. casei*, *L. acidophilus, L. rhamnosus*, *Lactobacillus reuteri*, *Lactobacillus fermentum*, *Lactobacillus helveticus*, and *Lactobacillus johnsonii*) where the binding occurred specifically at teichoic acids on the peptidoglycan layer (Serrano-Niño et al., 2015). Due to that, it could explain the high binding efficiency of both live Lcs cells and cell wall fractions found in the present study. Other studies, however, showed that heat treatment increased the binding of AFB1 by lactobacilli (Ahlberg et al., 2015). It has been suggested that the disruption of the bacterial cell wall due the heat treatment caused the surface of the bacteria becomes available to form additional bonds with AFB1 (Assaf et al., 2017). In contrast, the heat treated Lcs cells demonstrated the lowest binding efficiency in this study. AFB1 is also known as a protein-binding compound (Wang et al., 2017). The heat treatment on Lcs may cause denaturation to the protein present at the surface of heat treated Lcs, thus render the protein structure to be bound by the toxin (Wang et al., 2017). Moreover, several studies have shown that the binding ability is mostly strain-dependent. This is highly due to the different composition of biochemical compounds on cell wall in all strains of probiotics.

#### Scanning Electron Microscopy (SEM)

**FIGURE 3:**
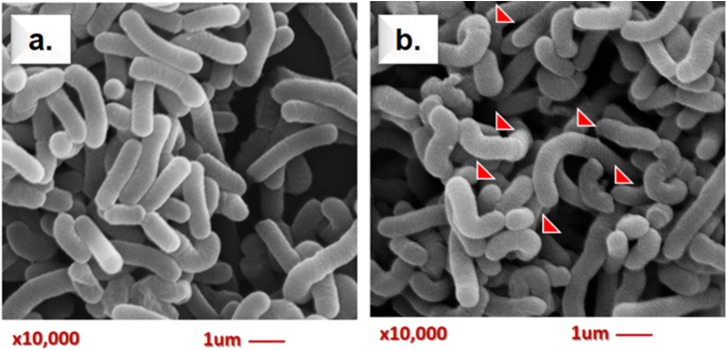
Scanning electron microscopy analysis of live Lcs cells + AFB1. **(a)** Control group (untreated Lcs). **(b)** Treatment group (Lcs incubated with 2 ug/ ml AFB1 for 1 h; 10,000 × magnification). Red arrows indicate structural changes induced by AFB1. The bacteria appeared irregular and rough on the surface as well as curve shaped.

Scanning electron microscopy assay is useful to capture the morphology alteration of bacteria by AFB1 treatment in this study. A representative micrograph of the normal morphology of Lcs (control) is shown in **Figure 3a**. The bacteria appeared undamaged and showed typical bacilli morphology. Meanwhile, the SEM micrograph of Lcs treated with AFB1 in **Figure 3b** revealed a prevalent conformational change. Such alterations in morphology were presumably caused by AFB1 bound on the cell wall surface. The most significant difference was the alteration in the shape of the Lcs. The appearance of curve-shaped Lcs was only found in the AFB1-treated sample. Furthermore, the bacterial morphology changes included irregular and rough surface.

The SEM analysis has some limitations as it is unable to determine the biochemistry composition of the bacterial surface. However, previous studies demonstrated that teichoic acid and β-D-glucan are found abundantly surrounding the bacterial cell surface of lactobacilli (Shetty and Jespersen, 2006). Lactobacilli are gram-positive bacteria, where the bacterial cell wall comprises of a layer of peptidoglycan and other molecules such as teichoic acids, polysaccharides (β-**D**-glucan), and proteins on the surface. Molecules such as salts, antibiotics, and divalent cations can cause structural changes on the teichoic acid structure (Palomino et al., 2013). Indeed, it has been reported that teichoic acid conformation may significantly affect the physiology of bacteria (Hernandez-Mendoza et al., 2010). Similarly, experimental data obtained by Hernandez-Mendoza et al. (2009b) demonstrated that teichoic acids may involve in AFB1-binding by Lcs. A molecular docking study performed by Yiannikouris et al. (2006) also found that the interactions between AFB1 and β-**D**-glucan of bacterial cell wall involved van der Waals interactions and hydrogen bonds. Furthermore, a study using atomic force microscopy approach reported surface structural changes on *L. reuteri* following the AFB1 treatment (Hernandez-Mendoza et al., 2011b). Hence, it can be postulated that the interaction between AFB1 and the surface teichoic acids as well as β-**D**-glucan structure of the cell wall of Lcs, may cause the structural changes observed in SEM micrograph (**Figure 3b**). These findings suggested that SEM might be a useful tool for the study of the AFB1-bacterial complex cell surface interaction. Further in-depth study using characterized compounds from Lcs coupled with molecular docking experiment (Yiannikouris et al., 2006) may reveal the significant role of cell surface compounds on the interaction with AFB1.

### *In Vivo* AFB1 Binding Assay

#### Body Weight Gain

**FIGURE 4:**
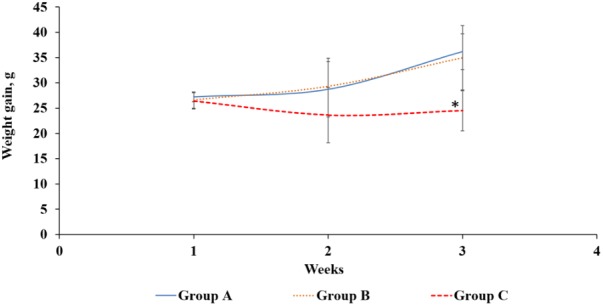
Body weight gain (g) of rats from three different groups throughout the treatment periods. Group A: control, oral gavaged with PBS only; Group B: oral gavaged with both Lcs and AFB1; Group C: oral gavaged with AFB1 only. Data are means from eight rats for each group (error bars indicate mean ± SD). An asterisk (^∗^) indicates a significant difference to group A and group B with *p* < 0.05.

Rats are good model to study AFB1-related toxicity as rats have been used to investigate the mechanisms of human aflatoxicosis. Rats exposed to AFB1 only (group C) consistently had a lower weight than the control (group A) and Lcs + AFB1 (group B), although the differences in weight between the treated and control group were statistically significant (*p* < 0.05) only at the end of the treatment (third week). Growth trajectory patterns showed deceleration in AFB1-exposed rats (group C). However, there was no significant difference between groups A and B regarding rats’ b.w. The comparison of rats’ b.w. gain between the three groups is shown in **Figure 4**. Based on the figure, animals in group A and B gained weight in a normal rate.

Similar to this finding, Hathout et al. (2011) demonstrated male rats fed with aflatoxins-contaminated diet showed a significant difference in b.w. compared with the control group and the group treated with probiotic (*L. reuteri* or *L. casei*). A study by Pawartha et al. (2015) demonstrated that the weight gain of male SD rats fed only Lcs was not significantly different from the healthy rats. However, the weight loss due to diarrhoea in the study was greatly reversed by Lcs treatment (Pawartha et al., 2015). The study indicates that the intake of Lcs will not lead to weight gain in normal condition. Based on previous studies, AFB1 may affect growth performance by causing liver dysfunction and anorexia, as well as via inhibiting lipogenesis and protein synthesis (Abbasi et al., 2018). Studies revealed that rats treated with AFB1 have lower leptin levels which reduced food intake, thus modulated energy balance and b.w. (Wang et al., 2010; Hernandez-Mendoza et al., 2011a). Low leptin level induced higher production of cortisol and interleukin-6 in the body. Cortisol and interleukin-6 are responsible for the feeding mechanisms, thus a high level of these compounds may lead to weight loss (Wang et al., 2010). Besides, AFB1 ingestion also affects digestive enzymatic activities and causes malabsorption syndromes (He et al., 2018). The formation of AFB1 metabolites such as AFB1-8,9- epoxide bind to DNA and proteins, which subsequently affect the enzymatic processes such as Krebs cycle, protein, and fatty acid synthesis, as well as gluconeogenesis (Jha et al., 2014). In addition, children exposed to AFB1 have been found to be associated with stunted growth (Ladeira et al., 2017). In this study, reduction of weight gain was diminished in Lcs-supplemented rats. This indicates Lcs might reduce AFB1 absorption in the intestinal tracts in group B. Based on the results on b.w. changes during treatment, it can be postulated that Lcs could alleviate the toxicity of AFB1 on b.w. gain of animals.

#### Determination of Blood Serum AFB1 and Urinary AFM1

Upon AFB1 exposure, AFB1 is readily absorbed into the blood via the gastrointestinal tract (Galarza-Seeber et al., 2016). Therefore, in this study, level of blood serum AFB1 was measured in all groups in order to investigate the effectiveness of the probiotic treatment in reducing the AFB1 level. As expected, AFB1 was not detected in blood serum samples from the rats in group A. Results showed that AFB1 level in blood serum of group B was statistically significant (*p* > 0.05) lower than in group C (**Table 2**). Lcs treatment in AFB1-exposed rat has a significant effect in reducing AFB1 levels in the blood. The findings indicate that probiotic Lcs is capable to bind to AFB1 and reduces AFB1 absorption in the intestine. This reduces the availability of free AFB1 and subsequently decreases the transport of free AFB1 to the liver for the metabolism process.

**Table 2:** The concentration of AFB1 in blood serum of AFB1-exposed rats.

Parameter	AFB_1_ (ng/ml)	*p*-value
**Group**		
B	49.6 ± 8.05	0.000
C	88.12 ± 10.65	


*Results are expressed as mean ± standard deviation of mean (*n* = 8). Group B: Lcs + AFB1; C: AFB1 only. *p*-value was obtained from independent *T*-test.*

The ingested AFB1 absorbed into the blood will be transported to other organs in the body, especially liver. In the liver, AFB1 is metabolized by CYP450 enzymes and leads to the formation of AFM1, a hydroxylated AFB1 derivative. In this study, the presence of AFM1 was assessed in all groups. AFM1 was not present in the urine of control (group A). Comparing the urinary AFM1 level between group B and C, there was no statistically significant difference, although the mean urinary AFM1 level in group C is higher than group B (**Table 3**).

**Table 3:** The concentration of urinary AFM1 of AFB1-exposed rats.

Parameter	AFM_1_ (ng/ml)	*p*-value
**Group**		
B	0.0219 ± 0.00	0.139
C	0.0388 ± 0.02	


*Results are expressed as mean ± standard deviation of mean (*n* = 8). Group B: Lcs + AFB1; C: AFB1 only. *p*-value was obtained from independent *T*-test.*

A study by Nikbakht Nasrabadi et al. (2013) showed that Lcs was capable to reduce AFB1 blood serum level by 2.76%. In the present study, rats in group B were pretreated with Lcs for 1 weeks before AFB1 exposure. Supplementation of Lcs before AFB1 exposure in rats reduced the serum AFB1 level up to 43%. The introduction of probiotic Lcs into the gut maintains a healthy gut microbiota composition. Probiotic is well-known for its effect in maintaining intestinal health by several pathways, especially by increasing the population of beneficial microbes in the gut (Stanley et al., 2016). Gut microbiota has been found able to bind to AFB1 as reviewed by Liew and Mohd-Redzwan (2018). Therefore, pre-treatment of Lcs for the rats in the present study further enhanced the detoxification of AFB1.

#### Histopathological Examination

**FIGURE 5:**
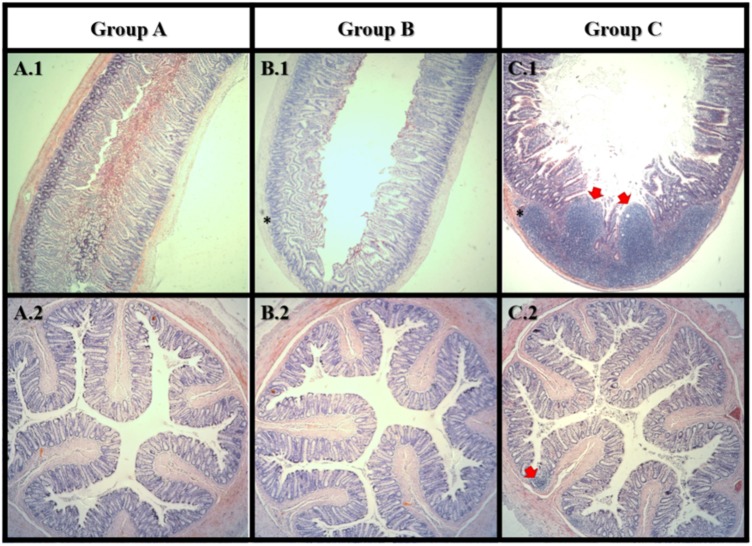
Haematoxylin and eosin staining of small intestine (1) and colon (2). Group A: control; Group B: Lcs + AFB1; C: AFB1 only. In small intestine, tumor-like growth (carcinoma) can be observed in group C only. Both group B and C showed lymphocytes accumulation (inflammation) in the small intestine. In colon, lymphocytes accumulation (inflammation) can be observed in group C only. Red arrow 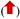 indicates tumor-like growth; Asterisks mark (^∗^) indicates lymphocytes accumulation. *n* = 8.

Histomorphologic changes in small intestine and colon H&E-stained sections following AFB1 exposure were evaluated in all groups in order to observe possible inflammatory responses or carcinogenicity. Representative micrographs of the small intestine (**Figures 5A.1,B.1,C.1**) and colon (**Figures 5A.2,B.2,C.1**) sections were shown to illustrate key observations. Histological analysis of H&E stained tissue revealed the small intestine and colon of group A was in a healthy state (5A.1).

After 4 weeks of AFB1 exposure, large carcinoma was observed in the small intestine of the AFB1-exposed rat (C.1) only. In the small intestine, lymphocytes accumulation was observed in both AFB1-treated (C.1) and Lcs + AFB1 (B.1) group. While in the colon, lymphocytes accumulation was only found in AFB1-exposed rats (C.2). Massive accumulation of lymphocytes indicates the occurrence of inflammation (Jacques and Elewaut, 2008). The results revealed the harmful effects of AFB1 toward small intestine and colon, and corroborated with a recent study (Nurul Adilah et al., 2018). The adverse effects of AFB1 toward small intestine is greater than colon. This may be due to the small intestine is the main site of aflatoxin absorption (Mohd Redzwan et al., 2016). Interestingly, few studies found intestinal epithelial cells can convert AFB1 into the reactive epoxide similar to hepatocytes. The ability is owed to the expression of CYPs by the intestinal epithelial cells. Exposure to dietary AFB1 has been associated with weight loss and environmental enteropathy such as histological changes in the small intestine includes abnormal growth and inflamed intestinal cells (Knipstein et al., 2015). Several studies have evaluated the toxicity of AFB1 in the intestine using colonic cell line (Caco-2). Zhang et al. (2015) demonstrated that AFB1 significantly (*p* < 0.05) increased apoptosis and lactate dehydrogenase activity besides causing genetic damage. The mechanism of AFB1 cytotoxicity is possibly via production of reactive oxygen species, causes damage to cell membrane and DNA. Besides, AFB1 reduced integrity of Caco-2 cells measured using transepithelial electrical resistance assay (Romero et al., 2016). Similarly, intestinal barrier function in broiler was affected by AFB1 (Wang et al., 2018). The adverse effects on the gut from AFB1 exposure include the disruption of intestinal barrier, cell proliferation, cell apoptosis, and immune system. This study demonstrated a prevalent AFB1-induced gut dysfunction as shown in **Figure 5** (C.1 and C.2). It is therefore suggested that gut absorptive and barrier functions of the animals should be monitored in future when using similar model.

*Lactobacillus casei* Shirota is well-known for its health promoting effect via its protective role in the gut. There is no significant of H&E images was observed between rats fed with Lcs alone and healthy control. (Pawartha et al., 2015). The presence of probiotic in the intestine can protect animals and human against xenobiotics, including AFB1 (Ahlberg et al., 2015). Besides preventing aflatoxicosis via direct binding toward AFB1, probiotic also excretes bioactive compounds. Bioactive compounds produced by probiotic have many beneficial properties including anti-inflammatory, antioxidant, anti-pathogen, anti-carcinogenic, and others (Di Cerbo et al., 2015). In addition, probiotics have been extensively tested in animal cancer models for their ability to prevent carcinogenesis, mostly in the intestine via short chain fatty acid production, modulating gene expression, as well as pH reduction (Zitvogel et al., 2015). Probiotic exerted its beneficial effects on health via modulating host immune system. Toll-like receptors (TLR) are the first line of immune barrier in the gut which responsible for recognition and differentiation of pathogen or non-harmful/ beneficial microbes (Loures et al., 2015). Both TLR 2 and 4 are important recognition agent for probiotic and contribute to the immunomodulatory effects of probiotic (Villena and Kitazawa, 2014). The findings in this study showed that AFB1 caused significant intestinal injuries. The presence of Lcs in the intestine provides a shield against AFB1-induced toxicity based on the histological observation. Intestinal adsorption plays an undeniably important role in preventing or reducing the systematic exposure of AFB1.

## Limitations and Recommendations

Even though our study found live cell and cell wall of Lcs exhibited the highest binding activity toward AFB1, the exact compounds which bind to AFB1 worth to be elucidated. For future study, it is recommended to evaluate the effect of pure compounds present in the cell wall of Lcs and other probiotics such as teichoic acid, peptidoglycan, phosphatide, membrane protein, and β-D-glucan toward AFB1 binding. Besides, the cause of alteration in bacterial cell surface after treated with AFB1 requires further investigation. This study lacks of a control group (rats that are fed with Lcs only), thus the direct effects of probiotic toward the normal rats were unclear. For future study, this control group should be included in the experimental protocol for better assessment and comparison between the groups. Generally, the administration of probiotics is beneficial to the rats which can be observed from the increment in b.w. and improvement of intestinal health. Such information can further confirm the mechanism on how the probiotics reduce the toxicity of AFB1.

## Conclusions

The Lcs cell components (live cell, heat treated cell, and cell wall fractions) have been shown to exhibit AFB1 removal ability. The results revealed that the Langmuir model is useful for the selection of the most efficient microorganism/compound for AFB1 removal by providing the binding efficiency parameters. Besides, the results obtained by microscopic techniques in this study may contribute to the better understanding of the interaction of bacterial cell wall components involved in the binding mechanism of AFB1. Regardless, extensive studies are required to simulate intestinal conditions in ex-*vivo* condition, in order to fully understand the mechanism of LAB in reducing intestinal absorption of AFB1.

*In vivo* experiment showed that AFB1 level was significantly reduced by Lcs through the binding process. Such effects were further confirmed through the lower occurrence of carcinoma and inflammation in small intestine and colon in the AFB1-exposed rat with Lcs supplementation. Nevertheless, inclusion of group fed with Lcs alone in this *in vivo* experiment can provide more information on the efficiency of Lcs in alleviating AFB1 associated symptoms.

Probiotic is a functional food that is growing in demand and its effect on health coupled with its ability to bind and remove aflatoxin is significant as one of the dietary approaches to prevent aflatoxin exposure and its adverse health effects. Taking into account the limitations of this study, findings from both *in vitro* and *in vivo* demonstrated that Lcs is a potential adsorbent of AFB1. Further studies with improved *in vivo* experimental protocol are warranted to elucidate the mechanism of AFB1 detoxification by probiotics.

## Author Contributions

W-P-PL, ZN-A, SM-R, and LT conceived and designed the experiments. W-P-PL and ZN-A performed the experiments. W-P-PL, ZN-A, SM-R, and LT analyzed the data. SM-R, and LT contributed reagents, materials, and analysis tools. W-P-PL and SM-R wrote the paper. SM-R and W-P-PL revised the article and approved the final version to be published.

## Conflict of Interest Statement

The authors declare that the research was conducted in the absence of any commercial or financial relationships that could be construed as a potential conflict of interest.
